# What interests young autistic children? An exploratory study of object exploration and repetitive behavior

**DOI:** 10.1371/journal.pone.0209251

**Published:** 2018-12-31

**Authors:** Claudine Jacques, Valérie Courchesne, Andrée-Anne S. Meilleur, Suzanne Mineau, Stéphanie Ferguson, Dominique Cousineau, Aurélie Labbe, Michelle Dawson, Laurent Mottron

**Affiliations:** 1 Psychoeducation and Psychology Department, Université du Québec en Outaouais, Gatineau, Québec, Canada; 2 Autism Research Group, CIUSSS du Nord-de-l’île-de-Montréal, Montréal, Québec, Canada; 3 Development Center, Centre Hospitalier Universitaire Ste-Justine, Montréal, Québec, Canada; 4 HEC Montréal, Montréal, Québec, Canada; 5 Psychiatry Department, Université de Montréal, Montréal, Québec, Canada; University Hospital of Tübingen, GERMANY

## Abstract

Behaviors characterized as restricted and repetitive (RRBs) in autism manifest in diverse ways, from motor mannerisms to intense interests, and are diagnostically defined as interfering with functioning. A variety of early autism interventions target RRBs as preoccupying young autistic children to the detriment of exploration and learning opportunities. In an exploratory study, we developed a novel stimulating play situation including objects of potential interest to autistic children, then investigated repetitive behaviors and object explorations in 49 autistic and 43 age-matched typical young children (20–69 months). Autistic children displayed significantly increased overall frequency and duration of repetitive behaviors, as well as increased specific repetitive behaviors. However, groups did not significantly differ in frequency and duration of overall object explorations, in number of different objects explored, or in explorations of specific objects. Exploratory analyses found similar or greater exploration of literacy-related objects in autistic compared to typical children. Correlations between repetitive behaviors and object explorations (their frequency and duration) revealed positive, not negative, associations in both groups. Our findings, from a novel situation incorporating potential autistic interests, suggest that RRBs do not necessarily displace exploration and its possibilities for learning in autism.

## Introduction

Behaviors, activities, or interests considered to be “restricted” and “repetitive” (RRBs) are one of two core domains which constitute the diagnosis of autism [1]. RRBs diagnostic of autism are diverse, encompassing motor mannerisms, repetitive use of speech or objects, insistence on sameness, atypical sensory responses, and intense interests, among others. In DSM-5 autism criteria, RRBs range from behaviors causing “significant interference with functioning in one or more contexts” (Level 1) to those which “markedly interfere with functioning in all spheres” (Level 3 [2]). In much of the literature, RRBs are viewed as detrimental to the progress and functioning of autistic individuals, as well as distressing to their families (e.g.[3–5]). For instance, an “optimal outcome” in autism, as currently defined, is in part achieved through loss of all but minimal, i.e. typical level, RRBs [6].

However, studies investigating associations between RRBs and various outcomes in autism have had mixed results [7, 8], including inconsistent associations between RRBs and measured intelligence depending on how RRBs are assessed [9]; no association between RRBs and adaptive behavior in “minimally verbal” children [10]; no association between RRBs and social-communication scores in preschool children [11]; and a positive association between intense (“circumscribed”) interests and non-verbal IQ (see also[12, 13]). RRBs did not predict language outcomes in speech-delayed autistics [14], and in early intervention studies, increased RRBs over time have been compatible with increased measured social abilities [15, 16] and increased measured intelligence [17]. The full range of evidence suggests that associations between RRBs and functioning in autism may be complex and not straightforwardly negative.

A particular concern has been that RRBs reduce autistics’ attention to and exploration of their environment, depriving them of meaningful input and opportunities for learning starting early in life [8]. Restricted object use, where exploration is confined to a small number and limited variety of objects, is considered to limit their learning opportunities [18, 19]. Sensory hyper- or hypo-reactivity and unusual sensory interests, which feature in DSM-5 RRB criteria, can all be seen as limiting exploration in autism [20–22], as can motor deficits [23]. Nevertheless, direct evidence of reduced exploration in autism, and its relationship with RRBs, remains sparse. Pierce and Courchesne [24] compared the object explorations of small groups of preschool autistic and age-matched typical children. Exploration was assessed in a room with 10 kinds of boxes and bags (e.g. “large shopping bag with handles wrapped in multicolored wrapping paper”), 6 of which contained an array of arbitrary objects (e.g. “a plastic sack filled with blue water and a plastic fish”). In this situation, where their potential interests were not considered, preschool autistic children showed decreased object exploration negatively correlated at a trend level with their repetitive behaviors. Using the same paradigm but with larger age-matched groups of toddlers, Bacon, Courchesne [25] again found fewer object explorations in autistics, as well as an altered quality of exploration rated as less appropriate and more stereotypical (e.g. “spinning wheels or visual inspection of objects”).

Among RRBs in autism, atypical or intense (“circumscribed”) interests may be the most commonly observed [13] yet have been the least studied across development. Evidence on relations among autistic interests, exploration, learning opportunities, and functioning remains sparse and disparate. In questionaires or interviews, the intense interests of autistic children were reported by their parents to cause functional impairment and great interference with activities [26, 27]; the intense interests of typical young children were, in contrast, positively perceived with no mention of interference even when such interests dominate children’s lives [28]. In eye-tracking studies using images of parent-reported general categories of autistic interests (e.g. vehicles), preschool autistic children showed an interest-driven pattern of visual exploration interpreted as circumscribed and preserverative [29, 30]. A similar study in older children and adolescents found that autistics visually explored fewer objects with longer fixations, but increased object exploration was correlated with increased RRBs [31]. In adolescents and adults performing a selective attention task, there is also preliminary evidence that in some circumstances autistics may experience less distraction or interference from their intense interests than do typical individuals [32]. This result was contrary to predictions and suggests the importance of improved and more direct tests of when or whether RRBs, which encompass atypical autistic interests, limit or interfere with exploration and attendant learning opportunities in autism.

In this exploratory study, we developed a novel stimulating play situation incorporating objects of potential interest to young autistic children. It was used to investigate whether object exploration in this population is reduced in duration, frequency, or variety and/or complexity of objects explored. We assessed the frequency and duration of overall repetitive behaviors and object explorations and analyzed how they were associated, without excluding the possibility that exploring objects is one thing autistics may do during repetitive behaviors. We also assessed specific repetitive behaviors, as well as how many different objects were explored and which ones. Using different levels of structure in the play situation, we investigated whether exploration occurred spontaneously or required structured support. Young autistic children, regardless of their developmental test scores, were compared to age-matched typical children.

## Method

### Participants

The full sample included 92 children, 49 autistic and 43 typical, aged from 20 to 69 months. Autistic children were recruited from the autism database of Riviere des Prairies Hospital (HRDP) in Montreal. Autism diagnosis was established by a multidisciplinary team of expert clinicians using a comprehensive assessment, DSM-IV-TR or DSM-5 autism criteria, and clinical best-estimate judgment; all autistic participants scored above Autism Diagnostic Observation Schedule (generic or second edition version) autism spectrum cut-offs. None of the autistic children had another primary DSM-based diagnosis or an identifiable genetic condition. Typical children were recruited from a local daycare center. A questionnaire completed by parents confirmed that children in the typical group did not exhibit suspected or diagnosed autism, developmental delays, or behavioral issues (questionnaires missing for 4/43 typical children). While autistic and typical children were matched on age and sex, their performance on Mullen Scales of Early Learning-MSEL (data available for 40 autistic and 40 typical children, [33]) differed, as expected (e.g.[34, 35]), with fine motor, visual reception, receptive language, expressive language, and composite scores significantly lower in the autistic group (all p’s <0.001). See Table 1 for full sample participant characteristics.

**Table 1:** Participant demographics, full sample.

	Autistic	Typical	p*
**Total full sample**	N = 49	N = 43	
**Age in months (SD)**	47.1 (10.49)	42.8 (13.65)	0.09
**Boys : girls**	38 : 11	33 : 10	0.54
**Full sample with available MSEL scores**	N = 40	N = 40	
**MSEL composite (SD)**	63.7 (19.14)	110.7 (16.90)	<0.001
**MSEL visual reception (SD)**	35.8 (17.42)	55.8 (11.82)	<0.001
**MSEL fine motor (SD)**	27.7 (10.86)	52.3 (12.69)	<0.001
**MSEL receptive language (SD)**	28.1 (12.47)	54.5 (11.18)	<0.001
**MSEL expressive language (SD)**	25.4 (11.61)	56.5 (12.25)	<0.001

MSEL: Mullen Scales of Early Learning.

* Age and MSEL: T-tests. Boys:girls: chi-square. MSEL composite are standard scores (mean 100, SD 15). MSEL visual reception, fine motor, receptive language, and expressive language are all T-scores (mean 50, SD 10)

### Procedure

Diagnostic assessments took place at the HRDP Autism Specialized Clinic. Written informed consent was obtained from each child’s parent. The screening questionnaire was administered to typical children's parents after the Montreal Stimulating Play Situation (MSPS, described below), which was administered by 2 psychoeducators with an expertise in autism. The child’s caregiver observed MSPS from behind a one-way mirror, while a psychoeducator was in the testing room with each child. Behaviors were recorded by a trained cameraman and coded by two raters blind to child diagnosis. A psychologist or psychoeducator administered MSEL, during which participants were usually accompanied by a parent or caregiver. The study was approved by Riviere des Prairies Hospital and Université du Québec en Outaouais research ethics committees.

### Measurement intruments

#### Autism repetitive behaviors repertoire

First, a questionnaire was developed which combined repetitive behaviors reported in the autism literature with those elicited from a survey of 10 autism professionals. This questionnaire included Likert-scale questions probing how frequently a preliminary list of 34 repetitive behaviors was observed, followed by open-ended sections enabling professionals to report additional repetitive behaviors and objects involved in RRBs such as restricted object use, as well as objects of potential interest to autistics, including but not limited to sensory interests. The questionnaire was completed by 61 autism professionals (occupational therapists: 9.7%; speech therapists: 9.7%; developmental pediatricians: 11.8%; psychiatrists: 11.8%; psychologists: 27.1%; psychoeducators: 27.1%; unidentified professionals: 2.8%) with 4–28 years of experience working with autistic toddlers and/or preschoolers, from the two specialized autism clinics of the Université de Montréal Autism Centre of Excellence (HRDP and CHU Sainte-Justine). The resulting repertoire of repetitive behaviors was then revised by three of the authors (SM, CJ, VC) to render each behavior suitable for computerized coding. The final repertoire included a list of 48 repetitive behaviors, each with an operational definition (see Autism repetitive behaviors repertoire, S1 Table).

#### Montreal simulating play situation

The Montreal Stimulating Play Situation (MSPS) is a protocol for assessing object exploration and repetitive behavior in young autistic children. It was developed through a 2-phase (development, experimental) multi-step process as follows.

**Development phase.** A preliminary MSPS was drafted based on 30 objects, including those of potential interest to autistics, elicited by the questionnaire completed by autism professionals. This version was piloted by 2 autistic boys (ages: 32 and 48 months) and 2 typical children, a girl and a boy (ages: 30 and 42 months, respectively); repetitive behaviors and object explorations were scored by two independent coders (SF and CJ). Pilot results were then discussed by four of the authors (LM, SM, MD, and CJ), three of whom were both researchers and clinicians. They recommended modifications on different aspects of the situation: reduction of play period durations, making objects more accessible for children of different ages, modification of researcher location in the testing room, and placing the caregiver behind a one-way mirror. Four objects were also added to the preliminary MSPS, for a new total of 34 objects (see S2 Table).

**Experimental phase.** A first sample (Sample A: N = 21 autistic and N = 24 typical children) was assessed using the MSPS-A, the first version of the MSPS, directly derived from the development phase. In addition to the 34 pre-identified objects, the testing room contained a child-size table, two chairs, and a large opaque box with a lid in which 11 of the 34 objects were placed at the start of MSPS. A trained cameraman recorded MSPS-A without interacting with the child.

After completion of MSPS-A by sample A, the protocol was revised by three of the authors (SM, CJ, VC). One object unexplored by children in both groups was removed (odor game), 7 objects were added (iPad, doll, baby bottle, picture dictionary, helicopters, trains, tracks) and one object was changed (remote-controlled dinosaur removed, due to many children in both groups being afraid of it, and replaced by remote-controlled car) for a final list of 40 objects, including 11 placed in the box at the start of MSPS (see S2 Table). In addition, the testing room was equipped with two remote-controlled cameras, which allowed the cameraman to record MSPS from outside the testing room. These modifications led to a revised version, MSPS-B. In the next step of the experimental phase, Sample B (N = 28 autistic and N = 19 typical children) was assessed using MSPS-B.

See S3 Table for sample A and B demographics, and S1 Fig for the testing room layout. Note that while MSPS-A and MSPS-B represent iterations in the multi-step development of MSPS, these largely similar versions involve the same approach to assessing repetitive behavior and object exploration in autism, and are encompassed henceforth by the single term MSPS.

#### MSPS administration

Assessment with MSPS takes approximately 30 minutes, divided into 4 play periods, in this order: free play 1 (5 minutes), semi-free play (5 minutes), semi-structured play (about 15 minutes), free play 2 (5 minutes). Room layout and object location at the start of MSPS were the same across all children.

In free play 1, each child could move freely in the testing room and play with objects of their choice (but note that 11 of the objects were in the box and thus not yet available), while the researcher remained seated in a corner of the room. Free play 1 also served to introduce participants to the testing room. This was followed by semi-free play, where children also played with objects of their choice (except for the 11 objects in the box) and moved freely, but when they played with an object, the researcher activated it or copied their actions. In these first two play periods, children were free to do what they wanted and no behaviors were stopped or redirected unless there was a risk of injury. However, these two periods were interrupted if the child was inactive for more than two minutes. In semi-structured play, all objects from the first 2 play periods remained available for exploration, but in addition the researcher presented the 11 objects in the box one at a time and in the same order to each child. The researcher manipulated or activated each presented object for a maximum of three repetitions, while the child could explore each presented object for a maximum of two minutes before the researcher introduced the next object and gently put aside the previous one. However, the child was free to again explore presented objects after they were put aside, and was also free to explore the other objects available in the room. Finally, free play 2 was the same as free play 1, except the child could now explore all the objects, i.e. those available from the beginning plus the 11 objects from the box presented during semi-structured play.

As noted above, the first two play periods could be interrupted (for free play 1 this kind of interruption occurred with 3 autistic and 2 typical children, for semi-free play in 3 autistic and 2 typical children) and the the semi-structured play period did not have a pre-set time limit, instead ending after all 11 objects from the box had been fully presented. For the final play period, a small number of children indicated at this point that they wanted to go back to their parents; thus 2 typical children did not start, and 4 autistic and 2 typical chidren did not finish, free play 2. Nevertheless, the duration of each play period did not significantly differ between autistic and typical children (free play 1: t(90) = .210, p = .41; semi-free play: t(90) = .804, p = .424; semi-structured play: t (90) = .204, p = .84; free play 2: t(88) = .107, p = .700). See S5 Table for duration (means and SDs, in seconds) of each play period for each group.

#### Scoring of repetitive behaviors and object explorations

The repetitive behaviors repertoire and list of objects were entered in the coding system (Observer XT 11) which allowed the simultaneous coding of frequency and duration of repetitive behaviors and object explorations directly into a computer. Object explorations and repetitive behaviors were coded independently, although sometimes a child can display both at the same time.

**Object exploration coding.** With 2 exceptions, objects (see S2 Table) were coded as explored when and while a child touched or manipulated it. The exceptions were balloons and bubble gun, which were activated by the researcher; these objects were therefore coded as explored when the child looked at the activated object.

**Repetitive behavior coding.** As in the DSM-5 [2], repetitive behaviors included in the repertoire could be atypical by their nature (e.g. hand flapping) or by their intensity (e.g. lining up objects) (see S1 Table). Repetitive behaviors were therefore defined so that each instance could be easily coded. Each repetitive behavior was coded when the child performed the behavior as defined in the repertoire, regardless of whether objects were or were not involved.

**Coding procedure and inter-rater agreement.** Videos were coded by two undergraduate students, whose knowledge of autism was probed via an interview and found to be incidental only and similar to what would be expected from the general population. The two coders were also naive to group status of each child and to the goals of the study. They were trained over multiple sessions by S.M., C.J., and V.C. to be able to identify behaviors included in the repertoire (see above, and S1 Table), and object explorations, and code them into Observer until they reached an agreement of 90%.

In the literature, inter-observer agreement is generally carried out on short sections of video (e.g. 8 minutes videos, 3 out of 30 minutes, 10 minutes videos [24, 36, 37]). However, in the present study, to have an equal representation of each play period, we conservatively chose to calculate inter-observer agreements for 30% of randomly selected videos of the entire duration of the MSPS. Percentage of agreement for frequency and duration of repetitive behaviors and object explorations was calculated by dividing the total number of agreements by the total number of agreements + total number disagreements and multiplying by 100 (Total agreement/(Total agreements+Total disagreements) x 100). Mean inter-rater agreement for frequency, defined as the two observers coding the same repetitive behavior or object exploration during the same time period (repetitive behaviors or object explorations overlapping), was 85.72% (76.66% - 96.57%), which is similar to the agreement obtained with simpler coding schemes (e.g. three-point scale and 8 behaviors coded [22, 38]). Mean inter-rater agreement for duration, defined as the identification of the onset and offset of a repetitive behavior or object exploration within the same 5 second by the two observers, was 82.10% (72.86% - 95.58%).

### Analyses

Analyses were first conducted for sample A (MSPS-A) and sample B (MSPS-B) separately, to verify whether the 2 MSPS versions were comparable. First, independent t-tests with an alpha of 0.05 were used to compare autistic and typical children on frequency and duration of overall repetitive behaviors and overall object explorations. Second, analyses were conducted to explore differences in specific repetitive behaviors and exploration of specific objects between groups. Bonferroni corrections were applied to this second set of analyses, with a conservative alpha level adjusted to 0.001, to prevent type I errors resulting from multiple comparisons [39]. Data screening procedures showed that the frequency and duration of repetitive behaviors and object explorations presented considerable variability (1a-Repetitive behavior duration: skewness values ranged from 2.187 to 10.296, kurtosis ranged from 5.247 to 106.000, 1b-Repetitive behavior frequency: skewness values ranged from 1.668 to 10.296 and kurtosis ranged from 3.656 to 106.000; 2a-Objects exploration duration: skewness values ranged from 1.022 to 8.272, kurtosis ranged from -.247 to 76.136, 2b-Objects exploration frequency: skewness values ranged from 1.263 to 7.122 and kurtosis from .961 to 62.036). Nonparametric tests were therefore used since the data were not normally distributed [40]. Mann-Whitney tests were performed to compare between-group differences in mean ranks for frequency and duration of specific repetitive behaviors, and for exploration of specific objects. Fisher’s exact test was used to compare between-group differences in the proportion of children who presented specific repetitive behaviors, and who explored specific objects.

We found largely similar results in sample A and sample B; these are reported in detail in S1 Appendix as well as S3 and S4 Tables. Data from participants in sample A and sample B were thus combined and the same series of analyses were conducted for the full sample. In addition, total number of different objects explored was compared between groups, as were correlations between object explorations and repetitive behaviors (frequency and duration) for the entire MSPS and its 4 play periods. For these multiple correlations, where the priority was finding any negative associations, alpha was set at 0.05. Finally, at the suggestion of a reviewer, we conducted analyses of object explorations within each group across play periods; these are reported in S2 Appendix.

## Results

All results are for the full sample assessed with MSPS

### Overall repetitive behaviors

**Fig 1:**
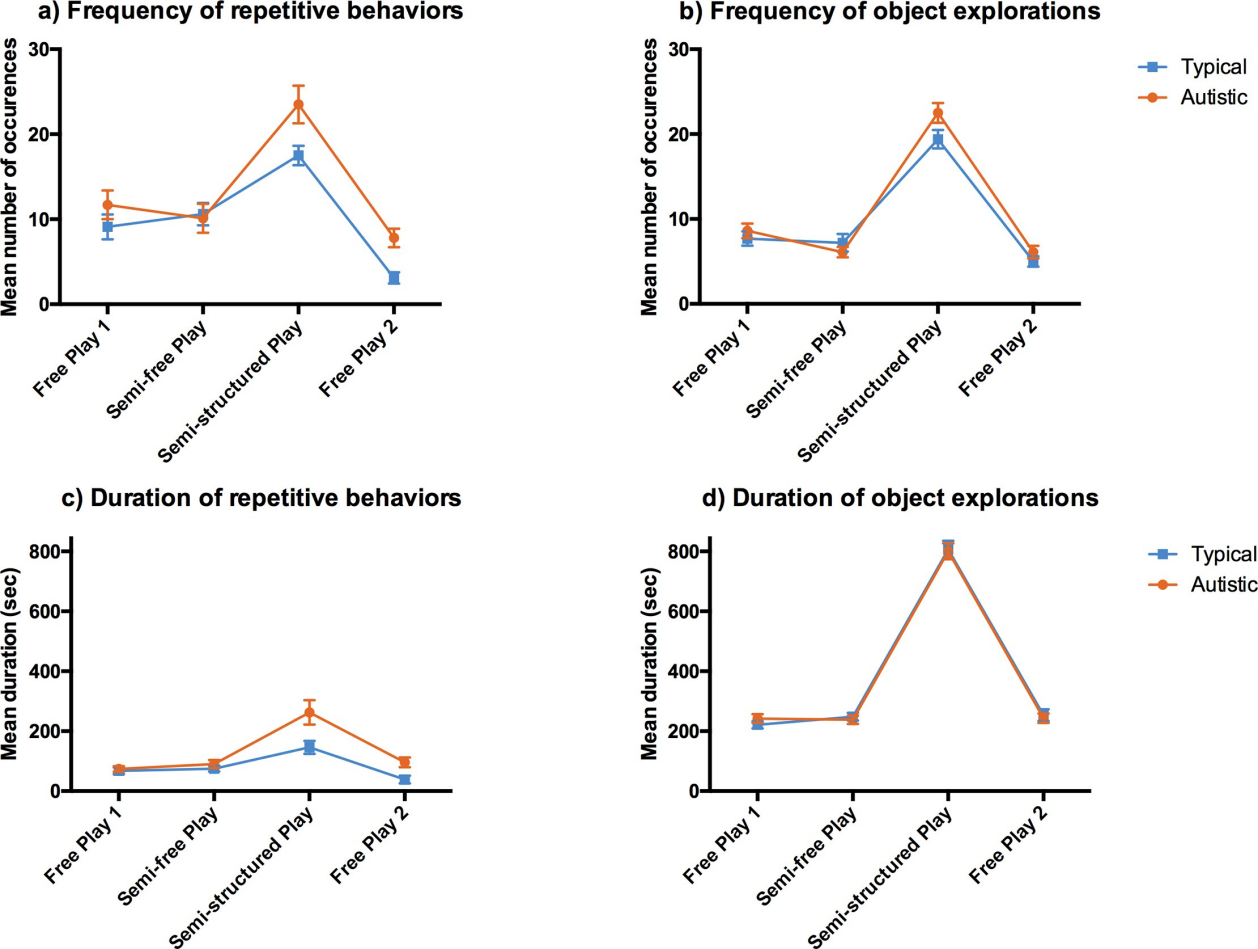
Frequency (number of occurrences) and duration (in seconds) of repetitive behaviors and object explorations in autistic and typical children across play periods, full sample. Error bars are standard error of the mean.

Autistic children displayed a significantly greater overall frequency (t (90) = -2.28, p = 0.03) and duration (t (90) = -3.38, p = 0.001) of repetitive behaviors compared to typical children during the entire MSPS. For the different play periods, repetitive behaviors were significantly more frequent in semi-structured play (t (90) = -2,38, p = 0.019) and free play 2 (t (90 = -3.67, p<0.001); and lasted longer in semi-structured play (t (90) = -2,49, p = 0.014) and in free play 2 (t (90) = -2.79, p = 0.006) in the autistic group. See Fig 1, and also Table 2 for all results (means, SDs) for the entire MSPS and each of the four play periods.

**Table 2:** Overall repetitive behaviors and object explorations, full sample.

	Frequency of repetitive behaviors		Duration of repetitive behaviors		Frequency of object explorations		Duration of object explorations	
	autistic	typical	autistic	typical	autistic	typical	autistic	typical
**MSPS (all periods)**	54.8* (36.0)	40.8 (19.7)	615.7* (468.9)	349.5 (235.0)	40.7 (14.9)	36.1 (14.7)	1529.1 (267.4)	1529.8 (279.8)
**Free play 1**	11.7 (11.9)	9.1 (9.6)	73.7 (68.6)	67.6 (74.9)	8.6 (6.0)	7.7 (5.5)	242.2 (103.3)	221.5 (80.3)
**Semi-free play**	10.1 (11.9)	10.6 (8.6)	90.5 (94.3)	74.8 (63.2)	6.1 (4.3)	7.2 (5.7)	238.0 (96.3)	248.4 (84.8)
**Semi-structured play**	23.5* (15.6)	17.5 (7.4)	262.6* (285.3)	146.1 (140.7)	22.5 (8.2)	19.4 (7.2)	800.4 (183.7)	806.6 (189.7)
**Free play 2**	7.8* (7.6)	3.1 (4.2)	96.3* (114.0)	38.9 (81.6)	6.1 (5.1)	5.0 (3.9)	243.3 (110.2)	253.3 (124.7)

Duration is in seconds. All values are means (SDs)

* indicates significant group differences.

### Specific repetitive behaviors

After Bonferroni correction, none of the repetitive behaviors was observed in a significantly greater proportion of typical children. Two behaviors were observed in a significantly greater proportion of autistic children: hand flapping (autistic = 45.1%; typical = 4.7%) and arm movements (autistic = 33%; typical = 4.6%), p's≤0.001. The proportion of autistic children presenting the behavior close gaze at objects was marginally greater than the proportion of typical children (autistic = 73%; typical = 41.9%), p = 0.002. For the mean ranks duration and frequency of specific repetitive behaviors, none of the behaviors was significantly more frequent or lasted longer in the typical group. In the autistic group, hand flapping (U = 655.00) and close gaze at objects (U = 626.00) were significantly more frequent, p's≤0.001, as well as significantly longer lasting (hand flapping U = 657.00; close gaze at objects U = 662.00) p's≤0.001, while arm movements was marginally more frequent (U = 794.00, p = 0.001) and lasted longer (U = 798.00, p = 0.002). See Table 3 for the mean ranks of frequency and duration, and the proportion of children, for the specific repetitive behaviors for which a significant group difference was observed in the full sample.

**Table 3:** Specific repetitive behaviors for which significant group differences were observed with respect to frequency, duration, and/or proportion of children, full sample.

	Frequency^1^ (mean ranks)			Duration^1^ (mean ranks)			Proportion of children^2^ (%)		
RRBs	autistic	typical	p	autistic	typical	p	autistic	typical	p
**Hand flapping**	56.16	37.23	<0.001	55.89	38.85	<0.001	45.1	4.7	<0.001
**Arm movements**	53.43	40.47	0.001	54.46	40.51	0.002	33	4.6	<0.001
**Close gaze at objects**	56.73	36.56	<0.001	56.02	37.40	<0.001	73	41.9	0.002

1- Mann-Whitney U test

2- Fisher’s exact test

### Overall object explorations

There were no significant differences between groups in mean duration (t (90) = 0.01, p = 1.0) and frequency (t (90) = -1.50, p = 0.14) of overall object explorations during MSPS. There were no significant group differences in each play period for object exploration duration (free play 1 t (90) = -1.08, semi-free play t (90) = 0.56, semi-structured play t (90) = 0.16, free play 2 t (90) = 0.42) and frequency (free play 1 t (90) = -0.74, semi-free play t (90) = 1.1, semi-structured play t (90) = -1.98, free play 2 t (90) = -1.2), all p’s>0.08. See Fig 1, and Table 2 for all results (means, SDs) for MSPS and each of the four play periods.

### Explorations of specific objects

**Fig 2:**
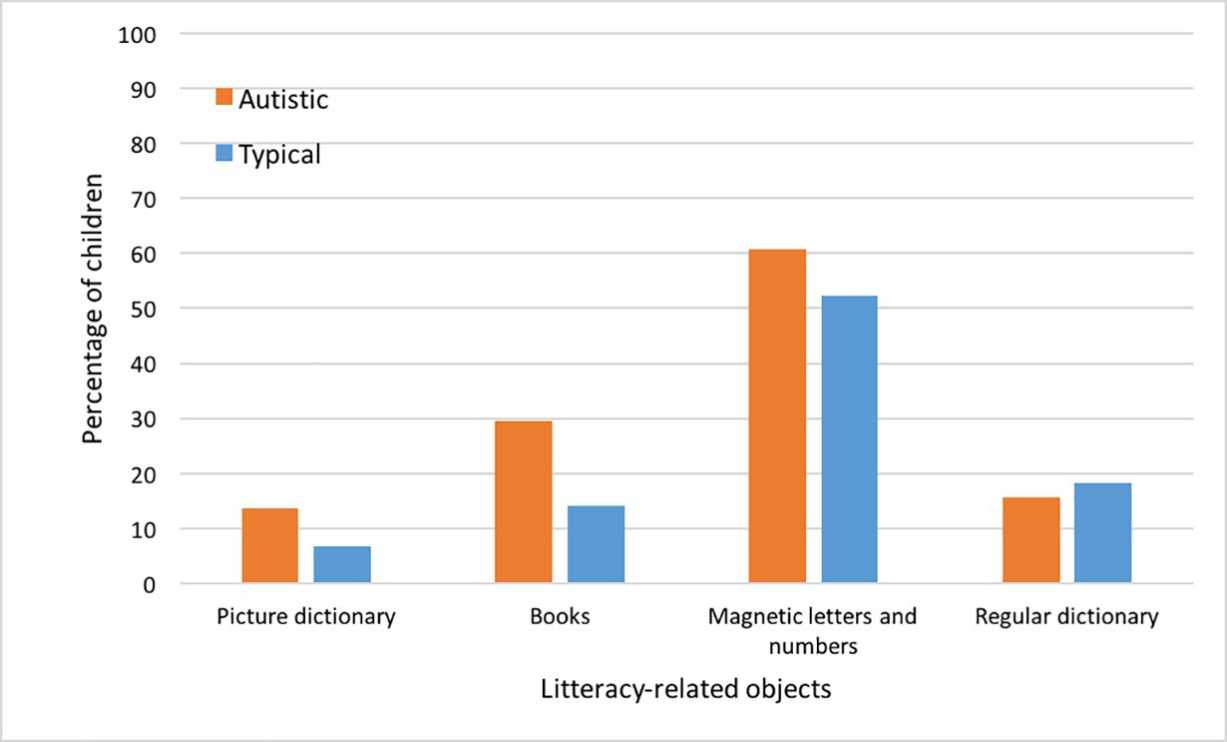
Proportion of autistic and typical children who explored objects related to literacy. Values represent proportion of children in percentage.

After Bonferroni correction, there were no group differences in explorations of specific objects. However, we investigated whether some objects where at the same time 1) explored by a greater proportion of children, 2) explored more frequently, and 3) explored for a longer time. None of the objects met all three criteria in the typical group, but a combination of greater proportion, frequency and duration was found for one object in the autistic group: books (proportion of children who explored books: autistic = 33% vs typical = 12%; frequency of exploration: autistic mean rank = 52.42 vs typical mean rank = 41.66; and duration of exploration: autistic mean rank = 51.80 vs typical mean rank = 42.40). We then looked more closely at all objects related to literacy (3 objects for the full sample, 1 object for sample B only). None of the group differences were significant following Bonferroni corrections. However, the proportion of autistic children who explored each object related to literacy was either similar to (one object) or higher than (3 objects) the proportion of typical children. See Fig 2, which shows data for the exploration of Regular dictionary, Magnetic letters and numbers, and Books from the full sample (N = 49 autistic and N = 43 typical children) and for the exploration of Picture dictionary from sample B only (N = 29 autistic and N = 19 typical children).

### Total number of different objects explored

In the entire MSPS, there was no significant difference between number of different objects explored by autistic (mean 17.8, SD 5.70) and typical (mean 18.8, SD 4.93, p = 0.377) children.

### Correlations between repetitive behaviors and object explorations

**Fig 3:**
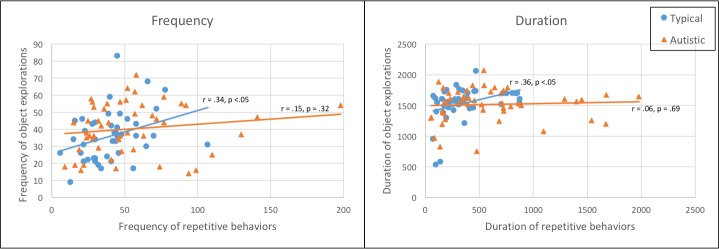
Scatterplots of frequency (number of occurrences) and duration (seconds) of repetitive behaviors vs object explorations in MSPS, full sample.

For the entire MSPS, frequency of repetitive behaviors was significantly and positively correlated with frequency of object explorations in typical (r = .336, p = .028) but not autistic (r = .146, p = .32) children. Similarly, duration of repetitive behaviors was significantly and positively correlated with duration of object explorations in typical (r = .358, p = .018) but not autistic (r = .058, p = .69) children. See Fig 3 for scatterplots of the data. The difference between groups was significant for duration (Z = 2.2, p = .028) but not for frequency (Z = .93, p = .35) correlations.

For the 4 play periods, correlations were either significantly positive (5 for autistic and 4 for typical children) or not significant (the remaining 7). Significant positive correlations were weak to moderate (r = .296 to .564). There were no significant negative correlations. See S6 Table for all correlations for MSPS and each of the 4 play periods.

## Discussion

In an exploratory study, we developed a stimulating play situation incorporating potential autistic interests. We assessed repetitive behaviors and object explorations in young autistic and age-matched typical children. Our findings confirm that compared to typical children, age-matched autistic children show a greater duration and frequency of overall repetitive behaviors, as well as increased specific repetitive behaviors. We did not, however, find significant group differences in object explorations, in frequency, duration, variety (number of different objects explored), or complexity (types of objects explored); nor did we find negative correlations between repetitive behaviors and object explorations. While our findings are provisional, they do not support concerns that repetitive behaviors reduce, limit, or interfere with object exploration in young autistic children.

We found RRBs were significantly increased in autistic compared to typical children, but the effects were small to medium (d = .476 for frequency; d = .706 for duration). That is, in a context including objects of potential interest, but where they were free to do what they wanted with respect to RRBs, young autistic children were not excessively preoccupied with RRBs, as might be predicted (e.g.[41, 42]). Autistic children showed some differences related to specific RRBs, with hand flapping, arm movements, and close gaze at objects being more frequent, of greater duration, and/or observed in a higher proportion compared to typical children. These results are consistent with observations throughout the history of autism, as well as with diagnostic criteria and instruments, and with numerous existing findings involving young autistic children (e.g.[38, 43–46].

Exploration of specific objects, which included all types of interaction with objects, did not significantly differ between groups in frequency and duration or proportion of children. Nor did autistic children require structure, as might have been expected (e.g.[37]), in order to explore objects in their environment: they did so spontaneously in free and semi-free play. Their exploration of complex material was not restricted even in an environment providing many simple or “sensory” objects.

Our sample size and use of strict correction for multiple comparisons made significant findings for specific objects difficult. However, acknowledging any possible lack of power to detect group differences in specific object explorations, we did perform exploratory analyses—considering the combination of higher proportion of children, frequency, and duration. Only one object, books, was more explored according to all three criteria by one group, and this was the autistic children. In addition, compared to typical children, autistic children showed similar or greater exploration of other literacy-related objects (e.g. picture dictionary, regular dictionary, magnetic letters and numbers). These findings are consistent with reported associations between autism and hyperlexia, which involves early intense spontaneous interest in written materials [47].

Our results, including those involving literacy-related materials, must be interpreted while considering that autistic children in this study were included regardless of their Mullen scores. They were age-matched with typical children whose mean Mullen scores were dramatically higher, all the more so in language domains. The autistic children in this study are thus the population that could be expected to be most excessively preoccupied by “lower level” RRBs, most confined to simple sensory interests (if any), and/or least exploratory [11, 42, 48, 49]. None of our findings supported these expectations.

Reducing RRBs in young autistic children continues to be an aim of various early autism interventions, including behavioral, developmental, parent-mediated, and pharmacological interventions (e.g.[41, 50–52]). However, the view that RRBs must be reduced for autistics to attend to their environment is not supported by our provisional results. A number of early autism interventions, including those considered “naturalistic,” feature efforts to make young autistic children’s non-social environments less interesting (e.g., rationing or removing access to interesting information, requiring that objects or materials be developmentally appropriate and typically used) in an effort to reduce RRBs and their interference with learning or progress (e.g.[53]). In one autism early intervention manual, the ideal room for intervention is described as having “nothing in it except a table and chairs and a closed or covered cabinet or shelves” (p.104 [50]). This and similar practices suggest that unless it is discouraged, as may be a direct or indirect aim of early autism interventions, exploration in autism is not reduced but increased, persistent, and thus disruptive to interventions with rigid procedures and objectives. Recent findings that school-aged autistic children show enhanced spontaneous visual attention to complex learning materials in their environment support this suggestion [54, 55]. A further speculative step, arising from our and other preliminary observations [56], may be asking what restricts autistic interests, that is, questioning to which extent autistic interests are restricted or circumscribed, and if so, why [57].

In this direction, the MSPS may in fact still underestimate the exploration, interests and potential of young autistic children. First, because most visual explorations included in the repetitive behaviors repertoire do not involve the manipulation of an object, they were not scored as object explorations, which they may plausibly be. Second, autistic children may benefit from more free play and less structured play, which in this study was the longest single play period, taking 15 of 30 minutes. Third, MSPS includes objects of potential interest to autistic children, but their individual interests are understudied and may be idiosyncratic [58]. Object selection was determined by surveying clinicians and searching the literature, where parent-report is prominent. While this led to the inclusion of some unusual but autism relevant objects, surveying autistic people, for example, may have produced a different list of objects with different perceptual or other characteristics (e.g., colors, sounds, topics, quantities, components). Object selection in MSPS is therefore open to improvement, possibly by including more in the way of complex objects, such as piano-type keyboards, which have been transformative for some young autistics [59], drawing materials [60], additional literacy-related materials [61], etc.

With respect to other possible limitations, we eventually hope to achieve sufficient power to compare repetitive behaviors and object explorations in relation to other variables of interest such as age, sex, and developmental level. For example, it would be important to investigate whether and how Mullen scores predict object explorations, repetitive behaviors, and associations between the two in young autistic children. We have also started to address concerns that the MSPS, as a novel situation full of new information, and free of efforts to reduce autistic behaviors, may cause negative emotions in autistic children. Preliminary data [62] suggest that these concerns are unfounded.

## Conclusions

We have provisionally found that in a context of some potential interest to them, young autistic children will display RRBs, but not to the detriment of exploration and its possibilities for learning. While many improvements are possible, this exploratory study has demonstrated the usefulness of an MSPS-type approach where a key element is giving autistic children free access to objects of potential interest to them, which they may spontaneously explore. Our results suggest that efforts to make autistic children’s environment more interesting—rather than efforts to reduce their repetitive behaviors—may lead to greater exploration, including of complex materials [63].

## Supporting information

S1 Appendix Results for sample A and sample B.
(DOCX)

S2 Appendix Within-group comparisons of object explorations across play periods, full sample. (DOCX)

S1 Fig Views of the testing room with objects. (DOCX)

S1 Table Autism repetitive behaviors repertoire. (DOCX)

S2 Table MSPS-A and MSPS-B object lists. (DOCX)

S3 Table Participant demographics, sample A and sample B.
(DOCX)

S4 Table Overall repetitive behaviors and object explorations, sample A and sample B.
(DOCX)

S5 Table Duration of play periods in seconds, full sample. (DOCX)

S6 Table Correlations between object explorations and repetitive behaviors (frequency and duration), full sample. (DOCX)
