# Supplementary material for: What interests young autistic children? An exploratory study of object exploration and repetitive behavior
Source: PLoS One. 2018 Dec 31;13(12):e0209251. doi: 10.1371/journal.pone.0209251 (PMC6312372; doi:10.1371/journal.pone.0209251)
Supplement: S1 Appendix — Results for sample A and sample B. (DOCX) [file pone.0209251.s001.docx]

**Supporting Information Appendix (Jacques et al.)**

**What interests young autistic children? An exploratory study of object exploration and repetitive behavior**

S1 Appendix. Results for sample A and sample B

**Supplemental appendix 1. Results for sample A and sample B**

**Sample A**

***Overall repetitive behaviors***

Autistic children displayed a significantly longer mean duration of overall repetitive behaviors than did typical children during MSPS-A (*t* (43) = -2.09, p=0.04). There was no significant difference between groups in frequency of overall repetitive behaviors (*t* (43)= -0.67, p=0.51). For the different MSPS-A play periods, repetitive behaviors lasted significantly longer in semi-structured play in autistic compared to typical children (*t* (43) = -2.18, p=0.03), with a similar result in free play 2 (*t* (43) = -2.40, p=0.02). However, repetitive behaviors were significantly more frequent in semi-free play in typical compared to autistic children (*t* (43) = 1.50, p=0.03). See Table S4 for all results (means, SDs) for MSPS-A and each of the four play periods.

***Specific repetitive behaviors***

After Bonferroni correction, there were no significant group differences in proportion of children displaying a specific repetitive behavior. However, one behavior, *hand flapping,* was presented by a marginally greater proportion of autistic (31.8%) than typical (0%) children (p=0.006). There were also no significant differences between groups in mean ranks for duration and frequency for any specific repetitive behavior, with *hand flapping* again only marginally longer-lasting (U= 150.00, p=0.007) in the autistic group.

***Overall object explorations***

There were no significant differences between groups in mean duration (*t* (43) = -0.66, p=0.52) and frequency (*t* (43) = -0.56, p=0.58) of overall object explorations during MSPS-A. For all play periods, there were no significant differences between groups in duration (free-play 1: *t* (43) = -1.87, p=0.07; semi-free play: *t* (43) = 1.12, p=0.27; semi-structured play: *t* (43) = -0.55, p= 0.59; free play 2: *t* (43) = 0.07, p=0.95) and frequency (free play 1: *t* (43) = -0.723, p= 0.47; semi-free play: *t* (43) = 1.94, p=0.06; semi-structured play: *t* (43)= -1.12, p= 0.25; free play 2: *t* (43) = -0.51 p= 0.62) of object explorations. See Table S4 for all results (means, SDs) for MSPS-A and for each of the four play periods.

***Explorations of specific objects***

There were no significant differences between groups in exploration of specific objects.

**Sample B**

***Overall repetitive behaviors***

Autistic children displayed a significantly greater mean duration (*t* (45) = -2.78, *p*=0.008) and frequency (*t* (45) = -2.30, p=0.03) of overall repetitive behaviors compared to typical children during MSPS-B. Repetitive behaviors lasted significantly longer in semi-structured play (*t* (45= -2.09, p =0.04) and were significantly more frequent in free play 2 (*t* (45) = -3.54, *p*=0.001) in the autistic group. See Table S4 for all results (means, SDs) for MSPS-B and for each of the four play periods.

***Specific repetitive behaviors***

After Bonferroni correction, no specific repetitive behavior was presented by a greater proportion of typical children. However, two behaviors were presented by a significantly greater proportion of autistic children: *hand flapping*: (autistic=55.2%; typical= 8.7%) and *arm movements* (autistic= 41.4%; typical= 0%), *p'*s ≤ 0.001). The behavior *close gaze at objects* was presented by a marginally greater proportion of autistic children (autistic=72.4%; typical=34.8%, p=0.007). When comparing mean ranks, none of the behaviors was significantly more frequent or longer-lasting in the typical group. In the autistic group, *hand flapping* (U=181.50), *arm movements* (U=195.50), and *close gaze at objects* (U=152.00) were all significantly more frequent, p’s ≤ 0.001, while *hand flapping* (U=182.50) and *arm movements* (U=195.50) lasted significantly longer (*p*'s= 0.001) and *close gaze at objects* (U=171.00) lasted marginally longer (p=0.002).

***Overall object explorations***

There were no significant differences between groups in mean duration (*t* (45) = 0.78, p=0.44) and frequency (*t* (45) = -1.51, p=0.14) of overall object explorations during MSPS-B. For all play periods, there were no significant differences between groups in duration (free play 1: *t* (45)= 0.38, p=0.71; semi-free play: *t* (45) =-0.19, p=0.89; semi-structured play: *t* (45) =-0.66, p= 0.52; free play 2: (*t* (45) =0.47, p=0.64) and frequency (free play 1: *t* (45) =-0.35, p= 0.73; semi-free play: *t* (45) =-0.10, p=0.29; semi-structured play: (t (45) =-0.56, p=0.58; free play 2: *t* (45) = -1.06, p= 0.29) of object explorations. See Table S4 for all results (means, SDs) for MSPS-B and for each of the four play periods.

***Explorations of specific objects***

There were no significant differences between groups in exploration of specific objects.
