# Supplementary material for: What interests young autistic children? An exploratory study of object exploration and repetitive behavior
Source: PLoS One. 2018 Dec 31;13(12):e0209251. doi: 10.1371/journal.pone.0209251 (PMC6312372; doi:10.1371/journal.pone.0209251)
Supplement: S2 Appendix — (DOCX) [file pone.0209251.s002.docx]

**Supporting Information Appendix (Jacques et al.)**

**What interests young autistic children? An exploratory study of object exploration and repetitive behavior**

S2 Appendix. Within-group comparisons of object explorations across play periods, full sample

**Supplemental appendix 2. Within-group comparisons of object explorations across play periods, full sample**

At the suggestion of a reviewer, Wilcoxon rank sum tests were performed to see if the different levels of structure in the play periods modified the frequency or duration of object explorations within each group. Bonferroni corrections were applied to these sets of analysis with an alpha level adjusted to 0.004 and the semi-structured play period was recalculated on 5 minutes (frequency or duration of object explorations divided by 3) to have the same denominator as the other play periods.

Within groups across play periods, object explorations were marginally more frequent (Z=-2.858, p=0.004) in free play 1 than in free play 2 in autistic children. In typical children, object exploration frequency was higher in free play 1 (Z=-3.395, p=0.001), semi-free play (Z=-4.249, p<0.001) and semi-structured play (Z=-3.628, p<0.001) when compared to free play 2, and object exploration was of longer duration in semi-structured play than in free play 1 (Z=-2.922, p =0.003). In interpreting these comparisons, however, it is important to note that 11 objects were in the box in the first 2 play periods, and were only made available for exploration in the second 2 play periods (see Methods, and Table S2).
