# Supplementary material for: What interests young autistic children? An exploratory study of object exploration and repetitive behavior
Source: PLoS One. 2018 Dec 31;13(12):e0209251. doi: 10.1371/journal.pone.0209251 (PMC6312372; doi:10.1371/journal.pone.0209251)
Supplement: S1 Fig — (DOCX) [file pone.0209251.s003.docx]

**Supporting Information Figure (Jacques et al.)**

**What interests young autistic children? An exploratory study of object exploration and repetitive behavior**

S1 Figure. Views of the testing room with objects

**
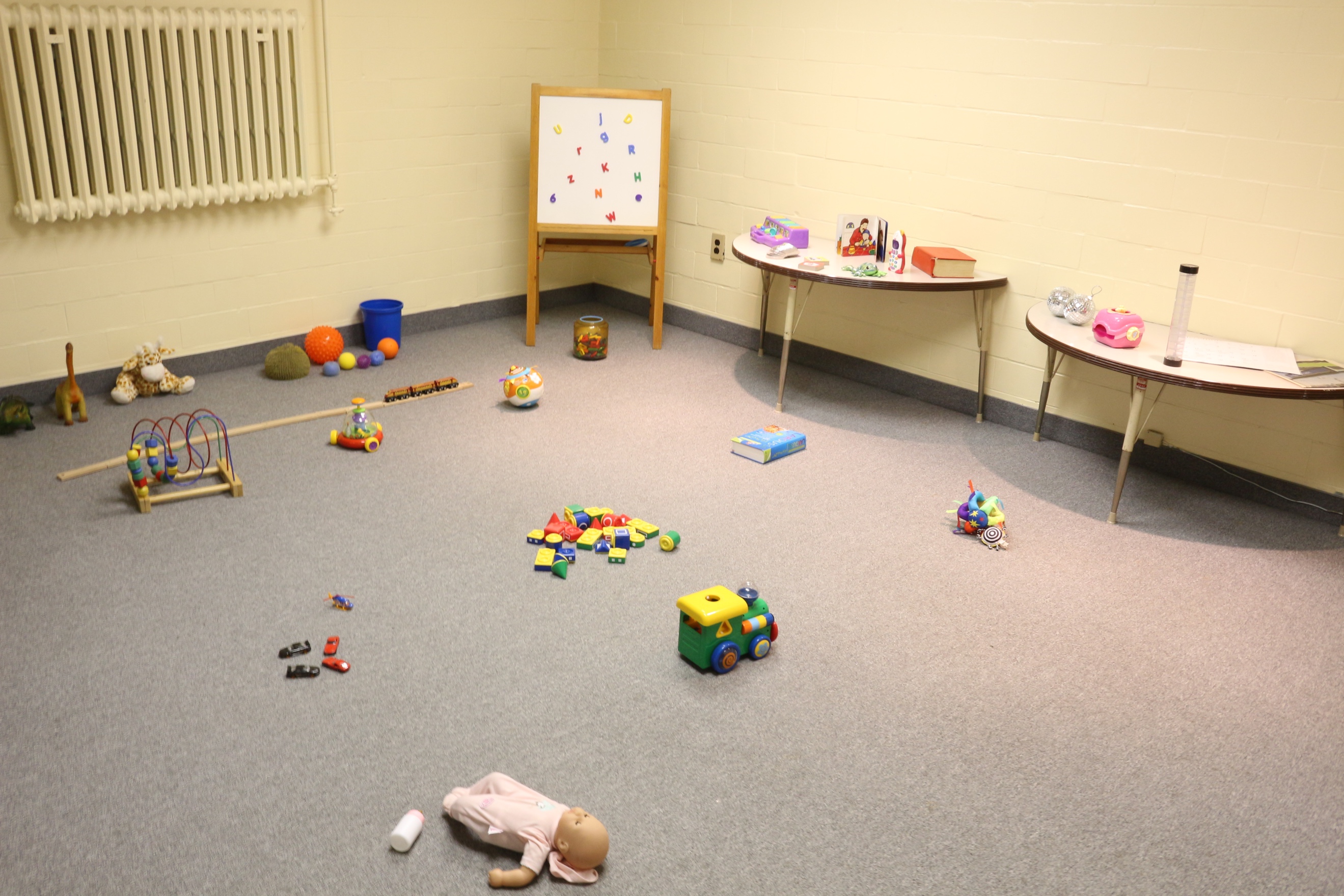
**
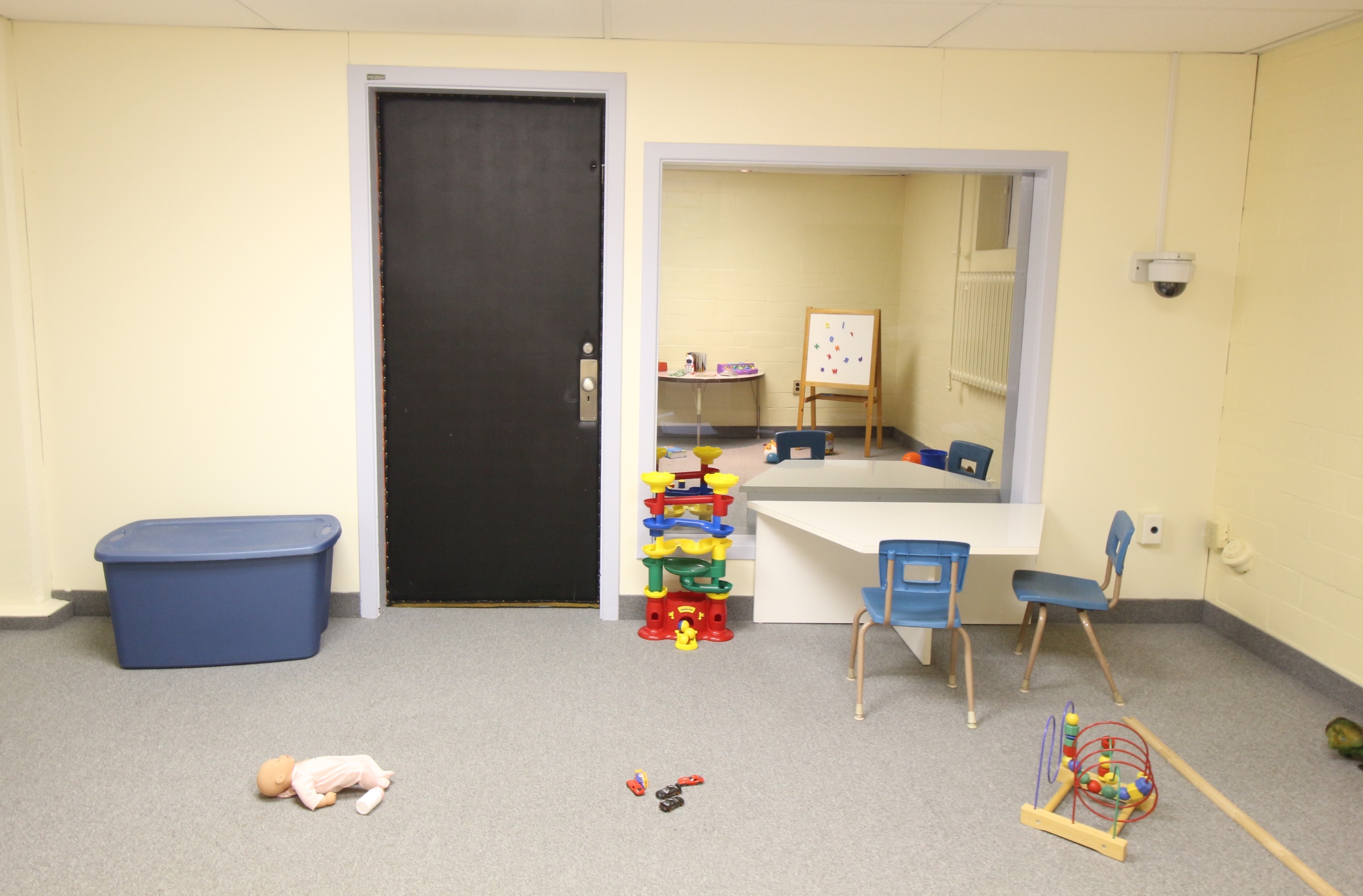


***Supplemental Figure 1*. Views of the testing room with objects**
