# Supplementary material for: What interests young autistic children? An exploratory study of object exploration and repetitive behavior
Source: PLoS One. 2018 Dec 31;13(12):e0209251. doi: 10.1371/journal.pone.0209251 (PMC6312372; doi:10.1371/journal.pone.0209251)
Supplement: S1 Table — (DOCX) [file pone.0209251.s004.docx]

**Supporting Information Tables (Jacques et al.)**

**What interests young autistic children? An exploratory study of object exploration and repetitive behavior**

S1 Table. Autism repetitive behaviors repertoire

**Supplemental table 1. Autism repetitive behaviors repertoire**

| **Behaviors** | **Description** |
| --- | --- |
| 1-Grouping objects by their perceptual properties | Regrouping objects by shape, color or texture |
| 2-Lining up objects | Alignment of objects; placing more than 2 objects in a symmetric pattern, in a line or in a shape (e.g., a square) |
| 3-Writing (without pencil) | Writing with plastic letters: a name, a word, or the alphabet (places at least 4 plastic letters) |
| 4-Pressing on object | Pushing on an object |
| 5-Holding object in hands | Holding one or several identical or different objects in one or both hands (threshold: more than 10 s) |
| 6-Putting object on ears | Placing one or several objects against the ear (other than the telephone) |
| 7-Putting object on cheek | Placing one or several objects on cheek |
| 8-Putting object in mouth | Placing one or several objects in mouth |
| 9-Dropping object | Dropping one or several objects |
| 10-Throwing object | Throwing one or several objects |
| 11-Turning object | Turning one or several objects around a vertical axis on a surface |
| 12-Rolling object | Rolling one or several objects in a circle on a surface |
| 13-Making object fly | Making one or several objects fly (without dropping it) |
| 14-Shaking object | Moving one or several objects up and down or side to side |
| 15-Stirring objects | Stirring objects with one or both hands or with another object |
| 16-Close gaze at object | Inspecting object by placing it 3 inches or less from the eyes |
| 17-Close gaze at fingers | Inspecting fingers or hands by placing them 3 inches or less from the eyes |
| 18-Lateral glances at object | Inspecting an object with a lateral gaze directed to the object in motion or still, either by turning head to the opposite side of the object, or by leaving the head straight and placing the object to the side of the face or body |
| 19-Lateral glances at fingers | Inspecting hands or fingers with a lateral gaze directed to the object in motion or still, either by turning head to the opposite side of the fingers/hands, or by leaving the head straight and placing the fingers/hands on the side of the face or body |
| 20-Obstructed gaze at object | Inspecting an object by partially blocking the view: either by closing one eye, or by half closing both eyes, or by placing an object between the eyes and another object |
| 21-Obstructed gaze at fingers | Inspecting fingers/hands by partially blocking the view: either by closing one eye, or by half closing both eyes, or by placing an object between the eyes and fingers/hands |
| 22- Blinking eyes | Opening and closing the eyes several times (at least 2 consecutive times) |
| 23-Facial twitching | Facial movement, squinting the eyes, the nose and the mouth at the same time |
| 24-Smelling object | Putting one or several objects under nose |
| 25-Finger in ears | Putting one or more fingers in ears |
| 26-Putting finger in mouth | Putting one or more fingers in mouth |
| 27-Putting part of body in mouth | Putting any part of the body in the mouth (except fingers) |
| 28-Covering ears | Hand or fingers touching the ears |
| 29-Hand and finger posturing | Stiffening of fingers of one hand or both hands (bent or outstretched) |
| 30-Wiggling fingers | Moves fingers with fast or repetitive movements |
| 31-Hand flapping | Flapping movements of semi-flexed hands and arms up and down and on each side of the body |
| 32-Clapping hands | Approaching hands following a movement similar to applauding |
| 33-Opening/closing hands | Opening and closing hands (threshold: after the third time) |
| 34-Hands rotation | Moving hands in a rotational motion |
| 35-Hands on eyes | Putting hands on eyes (threshold: after 10 s or at least 3 times) |
| 36-Arm movements | Moving one or both arms (implying the entire arm) |
| 37-Rubbing hands | Rubbing both hands together in backward and forward movements |
| 38-Rocking | Back and forth or side to side body movement |
| 39-Hopping | Jumping with feet together on the floor or on tiptoe (with feet on or off the ground) |
| 40-Spinning around | Spinning the whole body around on a spot |
| 41-Tiptoe walking | Walking from point X to point Y on tiptoe |
| 42-Pacing and running | Running back and forth from point X to Y |
| 43-Emits vocalizations spontaneously | Emits sounds (threshold: more than twice) |
| 44-Repeating sounds (echolalia) | Repeating sounds heard |
| 45-Emits words spontaneously | Emits words (threshold: more than twice) |
| 46-Repeating words (echolalia) | Repeating or tries repeating words |
| 47-Emits sentences spontaneously | Emits sentences (threshold: more than twice) |
| 48-Repeating sentences (echolalia) | Repeating sentence heard |
