# Supplementary material for: What interests young autistic children? An exploratory study of object exploration and repetitive behavior
Source: PLoS One. 2018 Dec 31;13(12):e0209251. doi: 10.1371/journal.pone.0209251 (PMC6312372; doi:10.1371/journal.pone.0209251)
Supplement: S2 Table — (DOCX) [file pone.0209251.s005.docx]

**Supporting Information Tables (Jacques et al.)**

**What interests young autistic children? An exploratory study of object exploration and repetitive behavior**

S2 Table. MSPS-A and MSPS-B object lists

**Supplemental table 2. MSPS-A and MSPS-B object lists. Objects in bold were in the box at the start of MSPS**

|  | MSPS-A | MSPS-B |
| --- | --- | --- |
|  | n=34 total  **n=11 in box** | n=40 total  **n=11 in box** |
| Abacus | √ | √ |
| Action-reaction game | √ | √ |
| Balls with lights and sounds | √ | √ |
| **Balloons** | **√** | **√** |
| **Big abacus** | **√** | **√** |
| **Boat: hammer and balls** | **√** | **√** |
| Books (3) with written texts | √ | √ |
| Baby bottle |  | √ |
| **Bubble gun** | **√** | **√** |
| Calendar | √ | √ |
| Cylinders with beads | √ | √ |
| Dinosaurs (2) | √ | √ |
| Doll |  | √ |
| **Elephant with throwing balls** | **√** | **√** |
| Empty bucket | √ | √ |
| Frog proprioceptive | √ | √ |
| **Game odors** | **√** |  |
| **Hoops** | **√** | **√** |
| i**-Pad: “The farm 1-2-3” application** |  | **√** |
| **Light and music stars** | **√** | **√** |
| Magnetic letters and numbers | √ | √ |
| Miniature cars (4) | √ | √ |
| Miniature helicopters |  | √ |
| Mirror balls (2) | √ | √ |
| Music box | √ | √ |
| Newspaper | √ | √ |
| Picture dictionary |  | √ |
| Regular dictionary | √ | √ |
| **Remote controlled car** |  | **√** |
| **Remote controlled dinosaur** | **√** |  |
| Slinky | √ | √ |
| Sound blocks | √ | √ |
| **Sound embedding objects** | **√** | **√** |
| Spinning light and sound | √ | √ |
| Stuffed giraffe | √ | √ |
| Tactile balls | √ | √ |
| Telephone sounds | √ | √ |
| Tracks |  | √ |
| Trains (3) |  | √ |
| Vibrating object | √ | √ |
| Visual and sound train | √ | √ |
| **Wheel caterpillar** | **√** | **√** |
