# Supplementary material for: What interests young autistic children? An exploratory study of object exploration and repetitive behavior
Source: PLoS One. 2018 Dec 31;13(12):e0209251. doi: 10.1371/journal.pone.0209251 (PMC6312372; doi:10.1371/journal.pone.0209251)
Supplement: S3 Table — Participant demographics, sample A and sample B. (DOCX) [file pone.0209251.s006.docx]

**Supporting Information Tables (Jacques et al.)**

**What interests young autistic children? An exploratory study of object exploration and repetitive behavior**

S3 Table. Participant demographics, sample A and sample B

| **Supplemental table 3. Participant demographics, sample A and sample B** | | | |
| --- | --- | --- | --- |
|  | **autistic** | **typical** | **p*** |
| ***Total sample A*** | N=21 | N=24 |  |
| **Age in months (SD)** | 47.0 (10.9) | 41.3 (14.3) | 0.158 |
| **Boys : girls** | 17 : 4 | 18 : 6 | 0.517 |
| ***Sample A with available MSEL scores*** | N=12 | N=21 |  |
| **MSEL composite (SD)** | 65.3 (22.7) | 107.0 (15.6) | <0.001 |
| **MSEL visual reception (SD)** | 33.4 (17.6) | 53.5 (11.1) | <0.001 |
| **MSEL fine motor (SD)** | 28.7 (12.8) | 50.0 (12.2) | <0.001 |
| **MSEL receptive language (SD)** | 29.2 (14.7) | 50.4 (10.1) | <0.001 |
| **MSEL expressive language (SD)** | 27.0 (13.1) | 54.7 (13.0) | <0.001 |
| ***Total sample B*** | N=28 | N=19 |  |
| **Age in months (SD)** | 47.2 (10.4) | 44.2 (13.2) | 0.367 |
| **Boys : girls** | 18 : 10 | 11 : 8 | 0.583 |
| ***Sample B with available MSEL scores*** | N=28 | N=19 |  |
| **MSEL composite (SD)** | 63.3 (17.1) | 114.1 (17.7) | <0.001 |
| **MSEL visual reception (SD)** | 38.2 (17.4) | 57.8 (12.3) | <0.001 |
| **MSEL fine motor (SD)** | 27.9 (10.7) | 54.5 (13.0) | <0.001 |
| **MSEL receptive language (SD)** | 27.8 (10.9) | 58.2 (11.1) | <0.001 |
| **MSEL expressive language (SD)** | 24.3 (10.1) | 58.2 (11.5) | <0.001 |

* Age and MSEL: T-tests. Boys:girls: chi-square. MSEL composite are standard scores (mean 100, SD 15). MSEL visual reception, fine motor, receptive language, and expressive language are all T-scores (mean 50, SD 10)
