# Supplementary material for: What interests young autistic children? An exploratory study of object exploration and repetitive behavior
Source: PLoS One. 2018 Dec 31;13(12):e0209251. doi: 10.1371/journal.pone.0209251 (PMC6312372; doi:10.1371/journal.pone.0209251)
Supplement: S4 Table — Overall repetitive behaviors and object explorations, sample A and sample B. (DOCX) [file pone.0209251.s007.docx]

**Supporting Information Tables (Jacques et al.)**

**What interests young autistic children? An exploratory study of object exploration and repetitive behavior**

S4 Table. Overall repetitive behaviors and object explorations, sample A and sample B

| **Supplemental table 4. Overall repetitive behaviors and object explorations, sample A and sample B. Duration is in seconds. All values are means (SDs); * indicates significant group differences.** | | | | | | | | |
| --- | --- | --- | --- | --- | --- | --- | --- | --- |
|  | **Frequency of repetitive behaviors** | | **Duration of repetitive behaviors** | | **Frequency of object explorations** | | **Duration of object explorations** | |
| **Sample A** |  | | | | | | | |
|  | **autistic** | **typical** | **autistic** | **typical** | **autistic** | **typical** | **autistic** | **typical** |
| **MSPS (all periods)** | 45.1 (31.2) | 39.8 (17.6) | 437.01* (351.5) | 265.12 (112.1) | 41.9 (12.9) | 39.7 (13.2) | 1519.7 (268.9) | 1459.3 (326.2) |
| **Free play 1** | 10.7 (12.0) | 6.9  (5.7) | 62.6 (66.7) | 46.8 (51.0) | 8.9  (6.2) | 7.6  (5.0) | 263.4 (113.4) | 205.8 (81.6) |
| **Semi-free-play** | 6.9  (6.9) | 12.3* (8.2) | 52.9 (45.3) | 73.9 (45.9) | 7.0  (4.1) | 9.8  (5.1) | 238.1 (97.0) | 268.0 (73.2) |
| **Semi-structured play** | 22.6 (15.0) | 17.1 (7.5) | 240.8* (253.0) | 112.4 (75.8) | 22.4 (7.7) | 19.5 (8.2) | 814.2 (171.5) | 233.0 (52.1) |
| **Free play 2** | 5.4  (5.0) | 3.3 (4.6) | 72.2* (97.2) | 16.9 (35.8) | 5.4  (3.8) | 4.9  (2.7) | 201 (94.9) | 203  (98.6) |
| **Sample B** |  | | | | | | | |
| **MSPS (all periods)** | 62.2* (38.0) | 41.7 (21.6) | 751.3* (505.8) | 422.9 (287.1) | 39.9 (16.4) | 33.1 (15.5) | 1536.2 (270.8) | 1591.0 (221.8) |
| **Free play 1** | 12.6 (11.9) | 10.8 (11.7) | 82.8 (70.0) | 87.0 (17.4) | 8.3 (6.0) | 7.8 (6.0) | 225.0 (93.0) | 234.1 (79.0) |
| **Semi-free play** | 13.0 (14.5) | 9.3 (8.8) | 122.3 (124.4) | 75.5 (102.3) | 4.4  (0.9) | 5.4  (1.1) | 237.9 (97.6) | 232.8 (91.5) |
| **Semi-structured play** | 45.1* (31.2) | 39.8 (17.6) | 437.0 (351.5) | 265.1 (112.1) | 41.9 (12.9) | 39.7 (13.2) | 1519.7 (268.9) | 1459.3 (326.2) |
| **Free play 2** | 9.7* (8.8) | 2.9* (4.0) | 115.9 (124.4) | 56.5 (102.3) | 6.6 (5.9) | 5.0  (4.7) | 277.5 (111.6) | 293.4 (130.6) |
