# Supplementary material for: What interests young autistic children? An exploratory study of object exploration and repetitive behavior
Source: PLoS One. 2018 Dec 31;13(12):e0209251. doi: 10.1371/journal.pone.0209251 (PMC6312372; doi:10.1371/journal.pone.0209251)
Supplement: S5 Table — (DOCX) [file pone.0209251.s008.docx]

**Supporting Information Tables (Jacques et al.)**

**What interests young autistic children? An exploratory study of object exploration and repetitive behavior**

S5 Table. Duration of play periods in seconds, full sample.

| **Supplemental table 5. Duration of play periods in seconds, full sample. All values are means (SDs).**   \|  \| Duration (seconds) \| \| \| --- \| --- \| --- \| \|  \| autistic \| typical \| \| Free play 1 \| 296.92 (62.58) \| 294.53 (43.31) \| \| Semi-free play \| 290.78 (60.4) \| 280.27 (64.8) \| \| Semi-structured play \| 921.53 (165.07) \| 928.42 (157.46) \| \| Free play 2 \| 312.06 (111.20) \| 309.84 (80.95) \| |
| --- | --- | --- | --- | --- | --- | --- | --- | --- | --- | --- | --- | --- | --- | --- | --- | --- | --- | --- |
