# Supplementary material for: What interests young autistic children? An exploratory study of object exploration and repetitive behavior
Source: PLoS One. 2018 Dec 31;13(12):e0209251. doi: 10.1371/journal.pone.0209251 (PMC6312372; doi:10.1371/journal.pone.0209251)
Supplement: S6 Table — (DOCX) [file pone.0209251.s009.docx]

**Supporting Information Tables (Jacques et al.)**

**What interests young autistic children? An exploratory study of object exploration and repetitive behavior**

S6 Table. Correlations between object explorations and repetitive behaviors (frequency and duration), full sample

| **Supplemental table 6. Correlations between object explorations and repetitive behaviors (frequency and duration), full sample** | | | | |
| --- | --- | --- | --- | --- |
| **Play Period** | **Group** | **Variable** | **r** | **p** |
| **Free play 1** | autistic | Duration | .564 | .000 |
|  |  | Frequency | .056 | .704 |
|  | typical | Duration | .348 | .020 |
|  |  | Frequency | .026 | .879 |
| **Semi-free play** | autistic | Duration | .289 | .051 |
|  |  | Frequency | -.028 | .854 |
|  | typical | Duration | .196 | .202 |
|  |  | Frequency | .162 | .294 |
| **Semi-structured play** | autistic | Duration | .336 | .022 |
|  |  | Frequency | .340 | .028 |
|  | typical | Duration | .152 | .306 |
|  |  | Frequency | .541 | .000 |
| **Free play 2** | autistic | Duration | .296 | .017 |
|  |  | Frequency | .434 | .002 |
|  | typical | Duration | .350 | .017 |
|  |  | Frequency | .464 | .001 |
| **Total MSPS** | autistic | Duration | .058 | .690 |
|  |  | Frequency | .146 | .320 |
|  | typical | Duration | .358 | .018 |
|  |  | Frequency | .336 | .028 |
